# ﻿Chromosome study of the Hymenoptera (Insecta): from cytogenetics to cytogenomics

**DOI:** 10.3897/compcytogen.17.112332

**Published:** 2023-11-01

**Authors:** Vladimir E. Gokhman

**Affiliations:** 1 Botanical Garden, Moscow State University, Moscow 119234, Russia Moscow State University Moscow Russia

**Keywords:** “Big data” approach, chromosome painting, fluorescence *in situ* hybridization, genome size, microdissection, whole genome sequencing

## Abstract

A brief overview of the current stage of the chromosome study of the insect order Hymenoptera is given. It is demonstrated that, in addition to routine staining and other traditional techniques of chromosome research, karyotypes of an increasing number of hymenopterans are being studied using molecular methods, e.g., staining with base-specific fluorochromes and fluorescence *in situ* hybridization (FISH), including microdissection and chromosome painting. Due to the advent of whole genome sequencing and other molecular techniques, together with the “big data” approach to the chromosomal data, the current stage of the chromosome research on Hymenoptera represents a transition from Hymenoptera cytogenetics to cytogenomics.

## ﻿Introduction

From the very introduction of the term “genome” (Winkler 1920), geneticists have been well aware that one of the most basic genomic features is how heritable matter of the nucleus is divided into separate cytological units, i.e., chromosomes. Moreover, the initial definition of this term, in fact, was indeed centered on the haploid chromosome set (Winkler 1920). Among organisms with sequenced genomes, insects play a crucial part due to their vast numbers and ecological significance (Li et al. 2019). Furthermore, they represent “an ideal group to examine the causes and consequences of chromosomal evolution. Insects are diverse with over one million named species, and are highly variable in chromosome number and in many other traits, such as sex determination systems, population sizes, generation times, habitats, and natural history” (Alfieri et al. 2023). This is also undoubtedly true for the largest insect taxa, e.g., Hymenoptera, which is one of the most species-rich, taxonomically complicated and economically important orders of insects. The current number of described members of this group exceeds 150 thousand (Huber 2017), and the potential number of Hymenoptera may well exceed a million species, mostly due to a large number of still undescribed parasitoids (Forbes et al. 2018). Among these insects, karyotypic data are available for just about two thousand members, and for many of them little is known beyond the chromosome number (Gokhman 2023), not to mention a few hymenopteran superfamilies (e.g., Orussoidea, Megalyroidea and Stephanoidea), for which karyotypes are completely unknown. Nevertheless, certain taxa, e.g., some parasitoids, ants and wasps, are apparently better studied in this respect than the others. In addition, molecular data on this order, including results of the whole genome sequencing, are also rapidly accumulating now (see, e.g., Branstetter et al. 2018). This paper briefly overviews the present state of cytogenetic research on Hymenoptera and discusses its place in the context of the genomic study of this vast group.

## ﻿Progress of the cytogenetic study of the order Hymenoptera

In a recently published review (Gokhman 2023), I have summarized the historical development of the karyotype research of the order Hymenoptera. According to this outline, three consecutive stages of this study took place in the 1890–1920s, 1930–1960s and 1970–1990s. Although chromosome research on this group was mostly done (and is still done today) involving traditional techniques, e.g., routine chromosome staining as well as C- and AgNOR-bandings, progressive accumulation of advanced methods did take place with time. This also applies to the current stage of karyotype research, which started in the 2000s (Gokhman 2023) with new techniques that involve both obtaining and analyzing primary karyotype data. Notably, a detailed description of the chromosome set of the honeybee, *Apismellifera* Linnaeus, 1758 (Apidae), appeared in the paper containing the first report of the fully sequenced genome of a hymenopteran (The Honeybee Genome Sequencing Consortium 2006). Nowadays, the number of species with sequenced genomes in the current version of the Hymenoptera Genome Database (https://hymenoptera.elsiklab.missouri.edu) (Elsik et al. 2016) approaches 120 (Walsh et al. 2022 onwards), i.e., it is approximately six times larger than the number of these species at the time of the first publication on this database (Elsik et al. 2016). However, the real number of sequenced genomes is much higher (perhaps more than 300), since many studied hymenopterans are apparently still not included into the database (see, for example, Gokhman et al. 2017 for information on the sequenced genomes of the members of the parasitoid genus *Aphelinus* Dalman, 1820 from the chalcid family Aphelinidae).

Estimates of the genome sizes obtained using cytometry and/or whole genome sequencing (e.g., Moura et al. 2020, 2021; Cunha et al. 2021b) can also provide some insights on the genome evolution within the order Hymenoptera. Specifically, a simultaneous analysis of the karyotypes and genome sizes of *Aphelinus* species (Gokhman et al. 2017) demonstrated that chromosomal rearrangements in this group usually occurred independently of the changes in the genome size. In addition, comparative studies of these parameters conducted on different populations of two of the three known species of the ant genus *Mycetophylax* Emery, 1913 (Formicidae), *M.conformis* (Mayr, 1884) and *M.morschi* (Emery, 1888), showed that conspecific populations were significantly different in terms of the genome size and total karyotype length despite having the same chromosome number and karyotype morphology (Moura et al. 2020). The authors of this study suggest that these changes in the amount of genomic DNA could represent initial stages of karyotype evolution within certain ant species.

Molecular methods have played a crucial role in the recent progress of chromosome research on Hymenoptera. While initial attempts to employ base-specific fluorochromes and fluorescence *in situ* hybridization (FISH) for studying karyotypes of this order date back to the 1990s (Odierna et al. 1993; Lorite et al. 1997), use of these techniques has greatly increased since that time. Specifically, staining with 4’,6-diamidino-2-phenylindole (DAPI) proved that the DNA that constitutes hymenopteran chromosomes is predominantly AT-rich (as in most eukaryotes), with the exception of nucleolus organizing regions (NORs), which are usually GC-rich and are therefore stained with chromomycin A_3_ (CMA_3_) (see, e.g., Bolsheva et al. 2012). Nevertheless, most chromosomes of a few bee and parasitoid species carry GC-enriched segments (mostly terminal ones; see Gokhman 2023 for review), and at least some of them definitely do not represent NORs. Ultimately, FISH with probes derived from either full or partial large transcriptional units of ribosomal DNA, e.g., 45S or 18S rDNA, can reliably visualize NORs on hymenopteran chromosomes (Bolsheva et al. 2012; Gokhman et al. 2014; Piccoli et al. 2018; Micolino et al. 2019; Menezes et al. 2021; Pereira et al. 2021; Teixeira et al. 2021; Cunha et al. 2023). FISH also demonstrated that heterochromatin contains repetitive sequences which often differ between related genera and species of Hymenoptera (Lopes et al. 2014; Cunha et al. 2020). Moreover, in this order different microsatellites can be characteristic either of heterochromatin or euchromatin (dos Santos et al. 2018; Piccoli et al. 2018; Travenzoli et al. 2019; Elizeu et al. 2021; Cunha et al. 2023). In addition, FISH can detect the presence of certain transposable elements on the chromosomes of parasitoid and aculeate Hymenoptera (Lorite et al. 2012; Li et al. 2017). Finally, certain unique sequences were also localized on hymenopteran chromosomes using FISH (e.g., Matsumoto et al. 2002).

Nowadays, karyotype evolution of many insect taxa, including Hymenoptera, can be traced using a number of powerful cytogenetic methods, e.g., microdissection and chromosome painting, which is also based on the FISH technique. Using these methods, Fernandes et al. (2011) demonstrated that in the karyotype of the bee *Tetragoniscafiebrigi* (Schwarz, 1938) (Apidae), centromeres of different chromosome pairs are heterogeneous in terms of their DNA content. On the other hand, Martins et al. (2013) explored B chromosomes of another bee species, *Partamonahelleri* Friese, 1900 using the same approach. These authors showed that a probe derived from a certain type of B chromosomes hybridizes only with these elements. In addition, Rütten et al. (2004), who used both microdissection and whole chromosome painting (WCP), were able to identify every chromosome in the haploid karyotype of the parasitoid, *Nasoniavitripennis* (Walker, 1836) (Pteromalidae) containing five metacentrics of similar size (n = 5).

Supergenes, i.e., tightly linked sets of loci that are inherited together, control complex phenotypes and are usually characterized by reduced meiotic recombination due to certain features of the genome, now play an increasingly important role in studying many aspects of ecology and genetics of various organisms (see, e.g., Berdan et al. 2022). Since inversions apparently represent the most frequent case of rearrangements responsible for restricting recombination between homologous chromosomes, it is not surprising that the first detected case of the supergene in the order Hymenoptera, namely, in the ant *Solenopsisinvicta* Buren, 1972, was explored, among other techniques, using cytogenetic analysis (Wang et al. 2013). In this species, a particular inversion was found to be responsible for the details of social organization of the colony, and similar rearrangements were later discovered in other members of the same family Formicidae (Brelsford et al. 2020; Lagunas-Robles et al. 2021; Kay et al. 2022; Chapuisat 2023) as well as in *Apismellifera* (Wallberg et al. 2017). We have recently found another putative supergene in two cryptic species of parasitoids of the *Lariophagusdistinguendus* (Förster, 1841) complex (Pteromalidae). These species have different chromosome numbers, n = 5 and 6, and a phylogenetic analysis based on molecular data indicates that chromosomal fusion occurred in this complex, with a certain acrocentric and a particular metacentric in the species with n = 6 corresponding to the shorter and longer arms of the largest metacentric chromosome in the species with n = 5 (König et al. 2019; Gokhman et al. 2019). This chromosomal fusion, together with a possible inversion in the longer arm of the above-mentioned metacentric in the species having n = 5, apparently prevents effective recombination between alternative variants of the supergene in these two morphologically indistinguishable species with strong biological differences (König et al. 2019). I therefore suggest that similar supergenes could also be responsible for the process of divergence of other groups of cryptic species of the order Hymenoptera.

A fascinating history of studying telomeric regions in the order Hymenoptera can serve as another example of applying a cytogenetic approach to the investigation of the genomic architecture of these insects. Specifically, these regions in most organisms have particular telomeric motifs; for example, the (TTAGG)_n_ repeat is characteristic of many insects (see, e.g., Kuznetsova et al. 2020). Although initial cytogenetic analysis apparently confirmed presence of this motif in Hymenoptera (Frydrychová et al. 2004; Vítková et al. 2005), only several dozen ant species as well as *Apismellifera* were studied at that time (Sahara et al. 1999; Lorite et al. 2002). However, the *Nasonia* Genome Working Group (2010) did not find this repeat in the genome of *Nasoniavitripennis*. Moreover, we also failed to reveal this motif on chromosomes of other studied parasitoids of the superfamilies Ichneumonoidea, Cynipoidea and Chalcidoidea (Gokhman et al. 2014). In addition, Menezes et al. (2013, 2017) showed that the (TTAGG)_n_ repeat is absent from the genomes of all studied aculeate Hymenoptera except for Apidae and Formicidae. Nevertheless, telomeric motifs in the suborder Symphyta remained unknown until the last five years, when we demonstrated presence of the canonical (TTAGG)_n_ telomeric repeat in two members of the sawfly family Tenthredinidae, thus suggesting the ancestral nature of this motif in the order (Gokhman and Kuznetsova 2018). Two years later, Dalla Benetta et al. (2020) finally identified the (TTATTGGG)_n_ repeat as the telomeric motif in *N.vitripennis* using both bioinformatic and cytogenetic approaches. Subsequent bioinformatic research has confirmed the two latter motifs, sometimes with a few variations, as characteristic features of the Symphyta and Chalcidoidea, respectively (Zhou et al. 2022). Furthermore, two recent studies (Fajkus et al. 2023; Lukhtanov and Pazhenkova 2023) have discovered an unprecedented diversity of telomeric repeats in the order Hymenoptera. Fajkus et al. (2023) demonstrated that short telomerase RNAs (TRs) in these insects are of the small nuclear RNA (snRNA) type, and are likely transcribed with RNA polymerase III. Surprisingly, this feature is characteristic of green plants and ciliates, apart from animals. Since TRs are used as templates for synthesizing telomeric motifs, the dramatic change in their structure and biogenesis have apparently led to an enormous increase in diversity of these repeats in the Hymenoptera. For example, TTAGGTCTGGG, TTGCGTCTGGG and TTAGGTTGGGG telomeric motifs were found in many aculeates, in the superfamily Vespoidea and in the genus *Bombus* Latreille, 1802 (Apidae) respectively (see also Lukhtanov and Pazhenkova 2023). On the other hand, Fajkus et al. (2023) did find the canonical insect repeat, (TTAGG)_n_, in a few parasitoids, including the only studied member of the family Mymaridae, thus confirming its basal position among other Chalcidoidea. Analogously, Lukhtanov and Pazhenkova (2023) detected the same motif in a number of bees (Anthophila) and in a few other aculeates, and showed that telomeric sequences in most insects represent arrays of short repeats interspersed by non-LTR retrotransposons, with those of the SART family prevailing in the Hymenoptera. Lukhtanov and Pazhenkova (2023) also hypothesize that insect telomeres are usually maintained by both telomerase-dependent and independent mechanisms, and shifts in the balance between these processes can lead to an increased diversity in the telomere structure as well.

The information summarized above also indicates that use of molecular data and availability of computational analytical tools provide new opportunities for analyzing karyotype information. This process has twofold significance. First, an increased computer power allows handling enormous amounts of chromosomal data (the so-called “big data” approach). Second, it leads to new, much more reliable phylogenetic reconstructions resolving many aspects of karyotype evolution. In the framework of the “big data” approach, for example, the chromosome number can be considered as a proxy for the level of recombination, and therefore its variation both among and within specific clades can point to different features of the evolutionary chromosome change. Indeed, a particular study of that kind was implemented about a decade ago on more than 1,500 members of the order (Ross et al. 2015). By calculating variance in the chromosome number in solitary vs. eusocial Hymenoptera, we demonstrated that this variance is about three times higher in the latter group, thus showing some specific features of the karyotype/genome evolution in the eusocial members of the order. Analogously, databases covering certain groups and/or particular chromosomal characters systematize our knowledge of the chromosome/genome features of the Hymenoptera and therefore help outlining pathways of the corresponding traits. These databases include the Bee Chromosome Database (https://bees.ufop.br) and the Ant Chromosome Database (https://ants.ufop.br) (Cardoso et al. 2018; Cunha et al. 2021a), as well as the databases on the number and position of ribosomal DNA (rDNA) clusters in animals (https://www.animalrdnadatabase.com) (Sochorová et al. 2021) and on the structure of telomere sequences, TeloBase (http://cfb.ceitec.muni.cz/telobase) (Lyčka et al. 2023). In addition, certain published reviews of chromosomal data of other large groups of Hymenoptera, e.g., Symphyta and Parasitica, are also available, although not in the form of online databases (Westendorff 2006; Gokhman 2009), but these publications are nevertheless substantially important.

The above-mentioned parallel accumulation of karyotypic and genomic data leads not only to general progress of cytogenetic studies of the Hymenoptera, but also to a qualitative transition toward a new level of cytogenetic knowledge, from studying separate DNA sequences to a network of interacting genes, and, ideally, to integral characteristics of whole genomes. On the other hand, this data accumulation allows independent checking of the results obtained by molecular and chromosomal techniques. For example, whole genome sequencing implies chromosome-level assemblies of different genomes, and counting chromosome numbers provides direct estimates of the numbers of linkage groups, which, in turn, can be compared to those of the obtained scaffolds.

Interestingly, all these features also characterize the newly introduced term “cytogenomics”. Although this term apparently lacks a universally accepted clear-cut definition, most experts agree that it implies a modern synthesis of cytogenetic and molecular approaches aimed at comprehensive research of the structure and functions of eukaryotic chromosomes with a special emphasis on DNA that constitutes these chromosomes (see, e.g., Liehr 2021). In addition, cytogenomics, which is sometimes also called “chromosomics” (Deakin et al. 2019), rather focuses on features of the entire karyotypes and genomes, as opposed to those of particular chromosomal regions and certain DNA sequences. However, since a considerable amount of information on Hymenoptera chromosomes is still obtained using classical cytogenetic techniques (see, e.g., Gokhman 2009), I argue that we are currently experiencing a transition from cytogenetic to cytogenomic research on Hymenoptera.

## ﻿Conclusions and future prospects

The present overview of cytogenetic research of the order Hymenoptera shows that, although many works still examine routinely stained chromosomes (see, e.g., König et al. 2019; Afonso Neto et al. 2022) and/or distribution and content of particular sequences and chromosomal segments, certain integral characteristics of the genomes are also studied. All this information suggests that we currently are in the process of transitioning from cytogenetics to cytogenomics of the Hymenoptera. As far as further prospects in cytogenomic research of Hymenoptera are concerned, I believe that they imply a combination of cytogenetic and molecular approaches, which will be focused on large chromosomal regions and whole chromosomes. Specifically, microdissection and chromosome painting could become powerful instruments of studying syntenies among hymenopteran karyotypes, especially in the case of complex rearrangements between closely related species. For example, chromosome sets of the two morphologically similar parasitoids of the genus *Anisopteromalus*, *A.quinarius* Gokhman et Baur, 2014 and *A.calandrae* (Howard, 1881), with n = 5 and 7 respectively, differ to an extent that prevents any feasible reconstruction of chromosomal rearrangements that led to the origin of those karyotypes (Gokhman et al. 1998). Under these circumstances, sequencing of microdissection products as well as use of other combinations of the cytogenetic and molecular approaches seem very promising. Finally, I am aware of only one use of specific antibodies to visualize particular components of hymenopteran chromosomes. Specifically, fluorochrome-conjugated antibodies showed the distribution of 5-methylcytosine along chromosomes of a certain parasitoid (Bolsheva et al. 2012), and I believe that similar studies could reveal many details of fine chromosome structure in this order.
